# Successful long-term monotherapy with rituximab in a patient with chronic lymphocytic leukemia of the B-cell-lineage: a case report

**DOI:** 10.1186/1752-1947-2-275

**Published:** 2008-08-14

**Authors:** Isrid Sturm, Joachim Oertel, Stephan Oertel, Jörg Westermann, Antonio Pezzutto

**Affiliations:** 1 Department of Hematology and Oncology, Charité Campus Virchow-Klinikum, Augustenburger Platz 1, 13353, Berlin, Germany; 2 Roche Pharma AG, Emil-Barrell-Str. 1, 79639, Grenzach-Wyhlen, Germany

## Abstract

**Introduction:**

Treatment of chronic lymphocytic leukemia of the B-cell-lineage is strongly based upon clinical staging because of the heterogeneous clinical course of this disease.

**Case presentation:**

We describe a 62-year-old patient with newly diagnosed chronic lymphocytic leukemia of the B-cell-lineage who did not respond to several chemotherapy regimens including chlorambucil, fludarabine and cyclophosphamide, developing a marked neutropenia and thrombocytopenia with life-threatening infections. Further chemotherapy appeared not feasible because of bone marrow toxicity. The patient was treated with 600 mg/m^2 ^rituximab weekly followed by eight courses of biweekly therapy and then by long-term maintenance therapy, achieving almost complete remission of the symptoms and disease control.

**Conclusion:**

After resistance to standard chemotherapy with chlorambucil and fludarabine, a patient with chronic lymphocytic leukemia of the B-cell-lineage was successfully treated with rituximab.

## Introduction

Chronic lymphocytic leukemia of the B-cell-lineage (B-CLL) is the most common form of adult leukemia and predominantly a disease of older individuals. Due to the strong heterogeneity in the clinical course of B-CLL with survival ranging from months to decades, treatment regimens are strongly based upon clinical staging. Although recent data show that cytogenetic profiling of tumor cells and flow-cytometry characterization of certain surface and intracytoplasmic proteins have strong predictive value, treatment regimens are still strongly influenced by clinical parameters such as clinical presentation, laboratory values or lymphocyte doubling time [1].

CLL is generally treated at the onset of symptomatic disease, and initial treatment includes alkylator therapy (chlorambucil or cyclophosphamide) or purine nucleoside analogs, such as fludarabine alone or in combination with cyclophosphamide. Rituximab is a humanized murine monoclonal antibody directed against the B-cell surface protein CD20 and is active against most B-lineage lymphoid malignancies, including CLL. Initially, the use of rituximab in CLL was considered unsafe because of severe toxic reactions from tumor cell lysis in patients with very elevated blood cell counts [2]. Here we describe a patient with B-CLL, who did not respond to prior chlorambucil and fludarabine chemotherapy developing a marked marrow toxicity to fludarabine, but was successfully treated with rituximab.

## Case presentation

**Figure 1:**
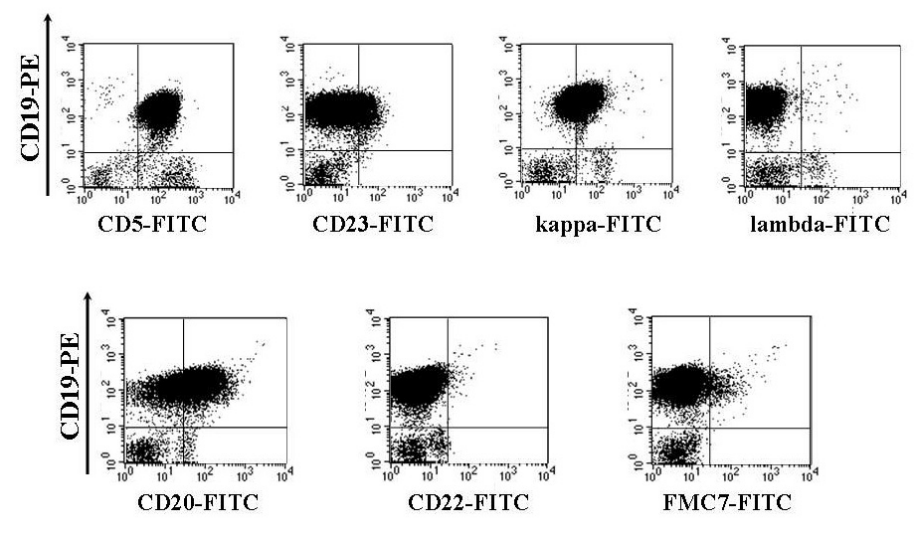
Immunophenotype of chronic lymphocytic leukemia of the B-cell-lineage at primary diagnosis in 2002: 61.7% of gated lymphocytes show CD20 expression.

A 62-year-old Caucasian woman (61 kg/160 cm) was first diagnosed with B-CLL stage Binet A/Rai 0 in July 2002. She showed marked leukocytosis with a white blood cell (WBC) count of 43.35 × 10^9^/l (82% lymphocytosis), accompanied by slight anemia (hemoglobin 11.1 g/dl) and a platelet count of 129 × 10^9^/l. Flow cytometric immunophenotyping (Fig. 1) of peripheral blood identified a CD5/CD19/CD20/CD23-positive clonal B lymphocyte population with kappa light chain expression and sIgM expression. CD10/CD103/CD38/CD43/CD22 and FMC7 were all negative. A chromosomal fluorescence in situ hybridization (FISH) analysis revealed del 14q32.

Upon this first presentation, the patient's general conditions were good, there was no peripheral lymphadenopathy or hepatosplenomegaly detectable, hepatic and routine laboratory parameters were in the normal range, plasma proteins as detected by electrophoresis and immunofixation were without pathological findings. As a consequence, a watch and wait approach was chosen.

Upon reevaluation in December 2002, a lymphocyte doubling time of less than 6 months was found, the patient had developed splenomegaly and cervical as well as axillary lymphadenopathy. The patient received five courses of chlorambucil until May 2003, but did not show significant regression of tumor lesions. Leukocytosis (230 × 10^9^/l) and nonhemolytic anemia (8.3 g/dl Hb) further deteriorated, accompanied by a 10% weight loss. Platelet counts remained normal at this time (174 × 10^9^/l). Administration of packed red cell transfusions was necessary. During the course of this first-line therapy, the patient developed a purulent bronchitis in March 2003 and shortly later, pneumonic pulmonary infiltrations.

**Figure 2:**
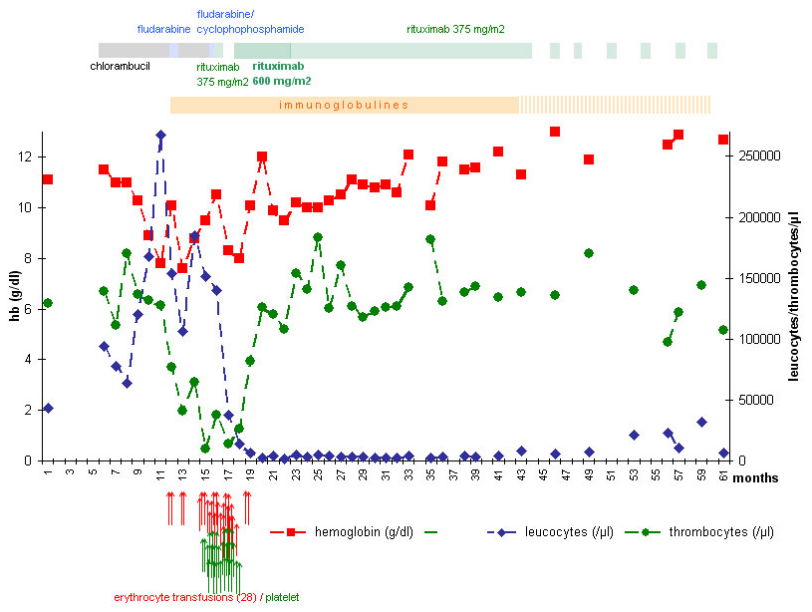
Course of hemoglobin, leucocyte and thrombocyte numbers under therapy with chlorambucil, fludarabine, cyclophosphamide and rituximab 375 and 600 mg/m^2^.

Fludarabine monotherapy was started in June 2003 at a dosage of 25 mg/m^2 ^day for 4 days. After the first cycle, the patient developed fever, a secondary antibody deficiency and a Coombs-positive severe anemia without detectable hemolysis. In addition, protracted thrombocytopenia developed. The patient became transfusion dependent for erythrocytes and platelets (Fig. 2).

Upon immunoglobulin substitution, the therapy with chlorambucil was reassumed. Until September 2003, the patient received three courses of chlorambucil (0.18 mg/kg for 10 days). Under this regimen, the CLL progressed to Binet stage C with subtotal bone marrow infiltration by mature lymphocytes with almost no residual hematopoietic activity (absolute granulocyte count 140/μl). Leukocyte count was high (210 × 10^9^/l). Bone marrow aspiration showed subtotal bone marrow infiltration with displacement of normal hematopoiesis. At this time, a fludarabine/cyclophosphamide (FC) combination chemotherapy was started. After the first cycle, the thrombocytopenia did not improve, and the patient experienced a retinal bleeding. There was no response toward CLL-related symptoms. As a further complication indeed, the patient developed a severe life-threatening abscessing pneumonia that was empirically treated with different broad-spectrum antibiotics including linezolid and additional amphotericin B. Cultures from sputum, bronchoscopy and blood were repeatedly negative.

Considering the remaining therapeutic options and the risks of any further aggressive chemotherapy in a severely pancytopenic patient, rituximab monotherapy was started with a weekly dose of 375 mg/m^2^. After a hospitalization of 35 days, the patient could be dismissed with improved clinical condition, still transfusion-dependent.

A few weeks later, pneumonia reactivation was diagnosed, accompanied by cholecystitis requiring hospitalization and new antibiotics treatment. Leukocyte count at this time was 20,000/μl with agranulocytosis (neutrophils 30/μl). Broad-spectrum antibiotics were given, and rituximab therapy was given at an increased dosage of 600 mg/m^2 ^weekly × 5, then 600 mg/m^2^biweekly × 8 without any other cytotoxic drugs.

During this therapy of intensified rituximab, the patient became transfusion-free. Twelve weeks after the first dose of 600 mg/m^2 ^rituximab, anemia abated to a hemoglobin-level of 9.5 g/dl, platelet count progressively rose to 136 × 10^9^/l and leukocyte count decreased to 5.08 × 10^9^/l. Upon normalization of platelets in April 2004, the rituximab dose was reduced to 375 mg/m^2 ^in a 4-week schedule while maintaining the immunoglobulin substitution. This regimen was sustained 20 times until November 2005. During this therapy, the hematopoiesis recovered completely, with the hemoglobin level reaching 11.9 g/dl, a WBC count of 4.38 × 10^9^/l, and a platelet count of 1.38 × 10^9^/l. Both the lymphadenopathy as well as the splenomegaly regressed completely. Over the entire term of maintenance therapy, treatment of pulmonary infections by oral antibiotics became necessary three times but could be performed at the outpatient level.

Due to the good condition of the patient, rituximab was tapered, with two administrations of 375 mg/m^2 ^in January and in April 2006. Since WBCs started to rise again 7 months after the last administration, rituximab maintenance therapy was reassumed in November 2006 at a dosage of 375 mg/m^2 ^every 3 months. The general condition of the patient remained good. In March 2007, hemoglobin was stable at 12.9 g/dl, leukocyte count was 11 × 10^9^/l (with 58% CD19+CD20+CD23+CD5+CD10-lymphocytes), and platelet count was 122 × 10^9^/l. With the exception of a minimal right-axillary lymphadenopathy, there were no pathological physical signs, and the Karnofsky Performance Scale score was rated 100%. Immunophenotyping of peripheral blood lymphocytes showed unchanged phenotype of leukemic cells that retained CD20 positivity. During the whole therapy, rituximab was tolerated well; a slow infusion rate was necessary on one or two occasions.

## Discussion

In this report, we describe the remarkable clinical response of a patient with rapidly progressive B-CLL and poor response to standard chemotherapy and with successful treatment with rituximab monotherapy. Because, so far, there is no clinical evidence favoring early chemotherapeutic treatment of B-CLL, therapy is not usually considered until evidence of disease progression is observed. With the patient presented here, there was no indication for treatment at first presentation, where we diagnosed B-CLL stage Rai 0/Binet A. According to current standards, treatment was delayed until a fast lymphocyte doubling time and rapid progression of tumor mass were observed. The general prognosis of this patient was unclear, as genotyping revealed a deletion in the V_H _gene locus. Translocations in this chromosomal region are frequently found in patients suffering from multiple myeloma and are associated with a poor prognosis. The meaning of del 14q32 mutations in B-CLL is not yet clear [3].

Although fludarabine as monotherapy or in combination with cyclophosphamide appears to be a highly effective regimen in CLL, many patients are still treated with chlorambucil as a first line therapy.

The complete lack of response to chlorambucil in our patient suggests an aggressive course of the disease, which was indeed documented by the rapid progression and deterioration of all hematological parameters.

In this setting, fludarabine is usually a valid therapeutic option. Although the incidence of autoimmune hemolysis or thrombocytopenia is well recognized, this therapy was accompanied by severe marrow toxicity, with virtual disappearance of normal leucocyte precursors and megakaryocytes on a bone marrow aspirate, as well as by a lack of efficacy. Although pulmonary infections are still a dangerous consequence of the secondary humoral immune deficiency in CLL, the frequency and severity of the infections in our patient were certainly due to a combined humoral and cellular immune defect.

The severity of the chemotherapy-induced marrow aplasia was surprising, and could be due to both a toxic effect, a lack of effect on CLL infiltration, or both. A further possibility would include a severe autoimmune reaction against all three lineages of hematopoiesis, including early precursors (i.e., amegakaryocytic thrombocytopenia).

In our opinion, further chemotherapy was particularly dangerous in this patient with extremely reduced general conditions, an active pulmonary infection and functional pancytopenia. Therapy with alemtuzumab was considered, but preference was given to rituximab because of the less severe compromise of the cellular immune system by rituximab, and because of the possibility that autoimmune phenomena might play a role in the functional marrow aplasia.

With rituximab monotherapy, response rates of 51% and 25% have been described as first-line treatment [4] and in patients with several pretreatments [5], respectively. The main drawback of rituximab monotherapy observed so far is the limited response to the induction therapy in pretreated patients [5]. Hainsworth and coworkers did report that patients with small lymphocytic lymphoma (SLL) and CLL who had shown an initial response or stable disease after rituximab induction therapy could be successfully retreated at 6-month intervals [4], but additional follow-up is required to fully assess the impact of this treatment strategy. Recently, we reported the efficacy and feasibility of a response-adjusted rituximab maintenance therapy in 12 patients with pretreated B-CLL [6].

It is important to underline that the response to rituximab became apparent when the dosage of the drug was increased to a much higher level, according to a publication by the Keating group [7] with dosages up to 2.25 g/m^2 ^in CLL patients. Both the weak expression of CD20 and the extremely elevated tumor burden of CLL patients (and certainly of the patient in this case) might explain the need for the higher dosage.

## Conclusion

Besides demonstrating the excellent response to rituximab in CLL, this case further suggests that maintenance therapy appears useful and feasible in CLL patients, which is in accordance with a recent report from our institution [6].

## Competing interests

Isrid Sturm, Joachim Oertel, Jörg Westermann and Antonio Pezzutto declare that they have no competing interests. Stephan Oertel is now employed by Roche company which is vendor of rituximab.

## Authors' contributions

IS, JO, SO, JW and AP were all involved in the diagnosis and treatment of the patient.

## Authors' note

In the meantime, after 4 years of disease control, the patient presented with progressive disease (abdominal bulk), and because CD20 expression was still present, rituximab treatment was intensified (375 mg/m2 weekly).
